# Phenotype and biochemical heterogeneity in late onset Fabry disease defined by *N215S* mutation

**DOI:** 10.1371/journal.pone.0193550

**Published:** 2018-04-05

**Authors:** L. Lavalle, A. S. Thomas, B. Beaton, H. Ebrahim, M. Reed, U. Ramaswami, P. Elliott, A. B. Mehta, D. A. Hughes

**Affiliations:** 1 Lysosomal Storage Disorders Unit, Department of Haematology, Royal Free Hospital and University College Medical School, London, United Kingdom; 2 UCL Institute of Cardiovascular Science, Barts Heart Centre, Barts Health NHS Trust, London, United Kingdom; 3 Haematology Department, St George’s Hospital NHS Foundation Trust, London, United Kingdom; The University of Tokyo, JAPAN

## Abstract

**Background:**

Fabry disease (FD) results from X-linked inheritance of a mutation in the GLA gene, encoding for alpha galactosidase A, and is characterized by heterogeneous clinical manifestations. Two phenotypes have been described “Classic” and “late onset” which cannot be predicted exclusively by genotype. The latter has been considered an attenuated form of the disease often affecting a single organ system commonly the heart. Recent studies have demonstrated that cardiac outcomes are similar in patients with classic and late onset mutations. In this study we investigate the relationship between clinical heterogeneity and plasma lyso-Gb3 in a large single centre cohort of *N215S* patients and compare this to patients with other mutations.

**Methods:**

In this single-centre, retrospective, cross-sectional study we analysed a cohort of 251 FD patients: 84 *N215S* mutation (37 males) and 167 *non-N215S* mutations (58 males). The Mainz severity score index (MSSI) was used as an index of overall disease severity. Cardiac function and morphology were assessed by electrocardiogram and echocardiogram. Left ventricular mass was calculated using the Devereux formula and the left ventricular mass index (LVMI) calculated to adjust for height (g/m^2.7^). The presence of white matter lesions was assessed by cerebral MRI or computed tomography (CT). GFR was measured by radio-isotope (chromium-EDTA) method and adjusted for patient height (ml/min/m^2.7^), and urinary protein quantification was undertaken by 24 hour urine collection. Plasma globotriaosylsphingosine (lyso-Gb3) was analysed prior to ERT in 84 patients.

**Results:**

*N215S* patients showed later symptom onset (males: p< 0.0001, females: p<0.03), later development of left ventricular hypertrophy (LVH) (median survival without LVH: 41 (*non-N215S*) vs. 64 (*N215S*) years, p< 0.0001), later development of proteinuria (median survival without proteinuria 43 (non-*N215S*) vs 71 years (*N215S*), p< 0.0001), later occurrence of cerebrovascular events (stroke/ Transient Ischaemic Attacks (TIA); median survival without stroke: 74 years (*non-N215S*) vs. not reached (*N215S)*, p< 0.02), later decline in renal function to GFR <60 ml/min/1.73m^2^ (median survival: 56 *(non-N215S)* vs. 72 *(N215S)* years, p< 0.01), and greater overall survival (median survival 81 *(N215S)* vs. 66 *(non-N215S)* years, p< 0.0006). Lyso-Gb3 was found to be less elevated in *N215S* compared to *non-N215S* male and female patients. However, the *N215S* population eventually reached an overall severity measured by MSSI comparable to the *non-N215S* without equivalent elevation of lyso-Gb3 (means: 6.7 vs. 74.3 nmol/L, p < 0.001). In addition, *N215S* patients showed strong correlations between lyso-Gb3 levels and LVMI, GFR, and MSSI. These associations became stronger when we investigated individuals’ life time exposure to lyso-Gb3 (calculated as [lyso-Gb3]*age): MSSI (r^2^ = 0.88, p< 0.0001), LVMI (r^2^ = 0.59, p< 0.005), and GFR (r^2^ = 0.75, p = 0.0001).

**Conclusion:**

These results demonstrate that the *N215S* mutation results in a late onset phenotype involving the heart and other organs. Correlations between clinical manifestations and plasma lyso-Gb3 variations in this group suggest a Fabry-relevant disease mechanism for the heterogeneity observed in this group.

## Introduction

Fabry disease (OMIM 301500; FD) is an X-linked metabolic disorder caused by mutations in the *GLA* gene [1], of which over 900 mutations (HGMD® July 2017) have been reported. The disease is characterized by deficient activity of α-Galactosidase A (α-Gal A) [1], and progressive multisystem deposition of its glycosphingolipid substrates [1, 2], including globotriaosylceramide (Gb3) and the deacylated form, globotriaosylsphingosine (lyso-Gb3) [3]. The accumulation process begins during fetal development [4] occurring in every organ [1], with particular deposition described in endothelial and smooth vascular muscle cells, leading to microvascular dysfunction [5]. Mutations of the *GLA* gene can be classified into three groups according to the resulting effect on the α-Gal A activity [6]: variants which result in an enzyme activity below 10% of normal in males (nonsense and certain missense mutations) [7], variants with residual enzymatic activity in the range of 15–30% (missense and certain splice *GLA* variants) [8] and variants which code for enzymes whose residual activity is not significantly reduced (normally about 35–40% of normal in males), also referred as non-pathogenic variants [6, 9]. Two phenotypes are distinguished “Classic” and “Later Onset”. The former has traditionally been associated with males and is characterized by an early onset, usually childhood, presenting with: periodic pain crisis (acroparesthesias), vascular cutaneous lesions (angiokeratomas), corneal and lenticular opacities, perspiration abnormalities, progressive proteinuric renal insufficiency, cardiac disease and cerebrovascular events. It is usually associated with mutations which result in very low (<1%) residual enzyme activity. The non-classic phenotype or later onset form, commonly involves a single organ system (usually cardiac or renal), and is generally associated with preservation of greater enzyme activity e.g. missense mutations [10]. This classification cannot be predicted exclusively by *GLA* variant as some mutations display both phenotypes [11, 12].

Clinical features of Fabry disease display significant heterogeneity in both men and women [1, 12]; heterozygous females have variable disease manifestations because of random X chromosome inactivation [10] and can manifest the full spectrum and severity of the disease as males, though on average a decade later [13]. Furthermore, variability in the clinical expression of the same mutation within members of a single pedigree and of unrelated pedigrees has been described [14, 15, 16]. It is possible that, as in other conditions, some of the heterogeneity is due to different genetic and immunological back ground or environmental profiles [17].

Plasma and urine Gb3 have been used as biomarkers of disease progression and treatment response [18, 19]. However, changes in Gb3 during the first year of therapy did not predict renal response [20]. More recently lyso-Gb3 has been proposed to better reflect the differences in phenotype of Fabry patients [21, 22]. Indeed, plasma lyso-Gb3 has been reported to be of greatest value in classical males with minimum elevation in females and later onset patients [23], and particularly higher in male patients with severe mutations, such as frame-shift or nonsense mutations [24]. As a non-invasive alternative, urinary lyso-Gb3 has also proved to be useful for diagnostics and has shown close correlation with genotype [25].

The *N215S* mutation is an A-to-G transition in codon 215 of exon 5, which causes the substitution of an asparagine by a serine (*N215S*) [26]. This mutation results in an obliteration of a functional N-glycosylation consensus site, but the enzyme is expressed at -5%-25% of normal activity in various tissues and cultured cells from affected patients [27]. In fact, it was first identified in an Italian patient as an atypical variant, who at age 42 had none of the classical Fabry manifestations (angiokeratoma, acroparesthesias, corneal or lenticular opacities, hypohidrosis, or renal insufficiency) but only manifestations confined to the heart [27]. Subsequently the *N215S* mutation was found to be common among atypical variants, presenting with either mild disease manifestations or asymptomatic [27, 28, 29]. Patients characterized in the relatively small study of 26 patients by Oder et al. lacked clinical manifestations commonly indicative of Fabry classical phenotype such as acroparesthesias, cerebrovascular events, chronic kidney disease (CKD), or angiokeratoma.

Despite the apparently isolated manifestations, cardiac outcomes for *N215S* patients and other cardiac variants are similar to that of classic patients [15, 30], and some *N215S* patients have shown more severe manifestation. Indeed, Oder et al. reported 3 out of 10 index subjects were diagnosed by renal glomerular biopsy after developing proteinuria of unknown origin. Furthermore, even though this mutation is predominantly associated with the later onset cardiac disease, heterogeneity in the phenotype is observed.

In an attempt to understand if this heterogeneity of severity and clinical manifestations is related to the Gb3 degradative pathway or other genetic or environmental factors we have tested the variation against circulating levels of lyso-Gb3 in a large single centre cohort of 87 *N215S* patients and compared this with other mutations. Our hypothesis therefore being that variation correlating with lyso-Gb3 implicates a Fabry-related disease mechanism.

## Materials and methods

The study received ethical approval by the Royal Free Hospital Ethics Committee and patients gave written, informed consent.

### Data collection

Retrospective case notes review was undertaken for all patients. Data was collected from baseline assessments regarding the route to diagnosis (family screening or index case), presenting symptom, baseline symptoms and baseline organ function. Screening of high risk populations, specifically cardiology clinics, was carried out. Family trees were constructed to determine the number of affected family members identified in each pedigree.

Decision making regarding the initiation of enzyme replacement therapy was undertaken according to national guidelines current at the time of baseline assessment.

Follow up data was collected regarding critical organ complications including: death, stroke, pacemaker/ implantable cardiac defibrillator (ICD) insertion and development of renal impairment or end stage renal failure (ESRF).

### Baseline assessments of organ function

Baseline assessment for the purposes of this study was defined as the date of initial comprehensive assessment of disease status undertaken at a specialist centre. Prior to the mid-1990s, few UK patients had a comprehensive assessment of their disease status (e.g. cardiac, renal and neurological function) and therefore in patients diagnosed prior to 1999 there is a lapse in time between date of diagnosis and baseline assessment. All baseline assessments were performed prior to initiation of Enzyme Replacement Therapy (ERT). A cross-sectional analysis of organ involvement at the time of baseline assessment was performed. This approach was taken so that this was not influenced by any effects of ERT on either organ manifestations or circulating lyso-Gb3 levels.

Cardiac function and morphology were assessed by electrocardiogram (ECG), and echocardiogram. Left ventricular mass was calculated using the Devereux formula [31] and the left ventricular mass index (LVMI) calculated to adjust for height (g/m^2.7^). Increased LVMI was defined as ≥48 g/m^2.7^ in females and ≥50 g/m^2.7^ in males. For assessment of proportion of patients with LVH both ECG and echocardiograms were considered, however, for Kaplan-Meyer analysis only LVMI by echocardiogram was included. Arrhythmia encompassed Wolf-Parkinson-White syndrome, supraventricular tachycardia, atrial fibrillation, ventricular fibrillation, non-sustained ventricular tachycardia, ventricular tachycardia and paroxysmal atrial fibrillation. Conduction abnormalities included long QT, short PR, and conduction blocks.

Quantification of urinary protein was undertaken by 24 hour urine collection and glomerular filtration was measured by radio-isotope (chromium-EDTA) method, adjusted for patient height (ml/min/m^2^). CKD was staged according to the Renal Association, UK.

The presence of white matter lesions (WML) was assessed by cerebral MRI or computed tomography (CT), and all images were reviewed by a neuroradiologist.

### Calculation of severity scores

Disease severity scores at baseline were calculated utilising the Mainz severity score index (MSSI) [32]. As severity increases with age, the baseline overall severity score was an age adjusted score, calculated by subtracting the calculated score from the predicted score for the patient’s age, as previously published [33].

We classified patients according to clinical severity, based on the MSSI, into “mild” (MSSI < 20), “moderate” (MSSI 20–40) and “severe” (MSSI > 40) phenotypes.

### Measurement of plasma enzyme activity and mutational analysis

All patients had mutational analysis performed and measurements of, either plasma or leukocyte, enzyme activity [34]. For mutational analysis, initial screening for abnormal exons was performed on leucocyte DNA by high resolution melt curve analysis. Sanger sequencing was then performed on abnormal exons to identify the causative mutation.

### Measurement of plasma Globotriaosylsphingosine

Samples for analysis of plasma lyso-Gb3 concentration were available for 169 patients (84 prior to commencement of ERT and 85 during ERT).

Plasma lyso-Gb3 levels were measured at the laboratory of Genetic Metabolic Diseases in the Academic Medical Centre using an (adjusted) tandem mass spectrometry method with glycine labelled as an internal standard [35].

In order to calculate life time exposure to lyso-Gb3 ([Lyso-Gb3]*age) we multiplied a patient’s lyso-Gb3 level by age at time of diagnosis.

### Statistical analysis

Statistical analysis was performed using Excel 2010, Microsoft® and GraphPad Prism version 5 (GraphPad®). For comparisons between two groups, t-tests were used for normally distributed data, Mann-Whitney U test if the data was not normally distributed and Fisher’s exact test to compare between proportions. Retrospectively collected data on events (development of stroke or transient ischaemic attack (TIA), development of stage III CKD (GFR <60ml/min/1.73m2), presence of WML, development of proteinuria, development of left ventricular mass indexed to height (LVMI) ≥ 50g/m2.7 in males and ≥ 48g/ m2.7 in females, and death) were used to assess the event free survival by using Kaplan-Meyer curves. Survival time was defined as the interval described in years between birth and event or last follow-up. A p value <0.05 was considered significant.

## Results

### Demographics

The cohort comprised 251 patients (95 male (37.8%) (current age: 15–87, median age 52 years), 156 female (62.2%) (current age: 12–90, median age 47 years)) from 96 different family pedigrees. Population characteristics are given in Table 1. Fifty-nine different mutations were identified. 67 patients (43 male, 24 female) were index cases and 184 (52 male, 132 female) were diagnosed on family screening. Eighty-four patients (33.5%) from 29 pedigrees (30.2%) had the *N215S* mutation. Most of these patients have been diagnosed in the 21^st^ Century, with only 3/84 patients (3.6%) of patients diagnosed prior to 2000 having the *N215S* mutation compared to 81/84 (96.4%) diagnosed 2000 onwards. 22/43 (51.2%) of index males had the *N215S* mutation.

**Table 1:** Demographics.

Parameters	N215S		non-N215S	
Overall	N = 84		N = 167	
	Males	Females	Males	Females
N (%)	37 (44.1)	47 (55.9)	58 (34.7)	109 (65.3)
**Age (years) at diagnosis**				
Mean (SD)	51.1 (19.2)	38.3 (16.1)	26.1 (16.4)	34.5 (16.8)
Median (range)	58 (13–74)	36 (11–78)	23 (1–65)	34 (4–75)
**Age (years) of symptoms onset**				
N (missing)	24^1^ (0)	14^2^ (0)	53^3^ (0)	59^4^ (4)
Mean (SD)	51.7 (14.5)	36.8 (21.6)	13.2 (12.6)	25.1 (16.4)
Median (range)	57 (7–73)	28.5 (13–66)	9 (1–57)	18 (5–61)
Index cases, n (%)	22 (59.5)	2 (4.3)	21 (36.8)	22 (20.2)
**First signs & symptoms:**				
Cardiac, n (%)	18 (81.8)	1 (50)	3 (14.3)	3 (13.6)
Renal, n (%)	2 (9.1)	1 (50)	1 (4.8)	1 (4.6)
Dermatological, n (%)	-	-	2 (9.5)	3 (13.6)
Neurological, n (%)	-	-	15 (71.4)	6 (27.3)
Stroke, n (%)	-	-	-	4 (18.2)
Ophthalmological, n (%)	-	-	-	4 (18.2)
Gastrointestinal, n (%)	2 (9.1)	-	-	1 (4.6)
**Leukocyte α-Gal A** (normal range = 33–134 nmol/mg protein/hr)				
N (missing)	15 (22)	14 (33)	31 (26)	47 (62)
Median (range)	7.0 (2.1–14)	49 (7–73)	2.3 (0.1–8.3)	33 (7.9–88)
**Plasma α-Gal A** (normal range = 4–21.9 nmol/ml/hr)				
N (missing)	36 (1)	46 (1)	49 (8)	102 (7)
Median (range)	0.2 (0–0.6)	4 (1.9–7.4)	0.1 (0–1.1)	3.7 (0.4–12.6)
**Overall severity (current),**				
N (missing)	35 (2)	43 (4)	49 (8)	94 (15)
Mild (MSSI < 20), n (%)	13 (37.1)	41 (95.4)	8 (16.3)	65 (69.2)
Moderate (MSSI = 20–40), n (%)	21 (60)	2 (4.7)	35 (71.4)	27 (28.7)
Severe (MSSI ≥ 40), n (%)	1 (2.9)	-	6 (12.3)	2 (2.1)
**Overall severity (baseline),**				
N (missing)	37 (0)	46 (1)	53 (5)	103 (6)
Mild (MSSI < 20), n (%)	19 (51.4)	42 (91.3)	6 (11.3)	73 (70.9)
Moderate (MSSI = 20–40), n (%)	17 (46)	4 (8.7)	45 (84.9)	29 (28.2)
Severe (MSSI ≥ 40), n (%)	1 (2.7)	-	2 (3.8)	1 (1)
**Age adjusted score**				
N (missing)	37 (0)	46 (1)	55 (2)	105 (4)
Mean (SD)	-5 (5.7)	-2.1 (5.4)	7.6 (0.9)	4.8 (7.1)
Median (range)	-6 (-13.9–6.5)	-2.5 (-11.7–9.2)	8.6 (-9.5–21.6)	3.7 (-7.6–20.9)
Currently on ERT, n (%)	33 (89.2)	11 (23.4)	56 (98.2)	62 (56.9)
Agalsidase alfa, n (%)	30 (90.9)	10 (90.9)	50 (87.7)	58 (93.6)
Agalsidase beta, n (%)	2 (6.1)	1 (9.1)	6 (10.5)	4 (6.5)

α-Gal A = alpha-galactosidase A; ERT = enzyme replacement therapy; Patients were stratified according to clinical severity, based on the Mainz Severity Score Index (MSSI), into “mild” (MSSI < 20), “moderate” (MSSI 20–40) and severe (MSSI > 40) phenotypes. Number of patients with no symptoms prior to diagnosis: 1. N = 13, 2. N = 33, 3.N = 4, 4. N = 46.

### Index case presenting symptoms

**Fig 1:**
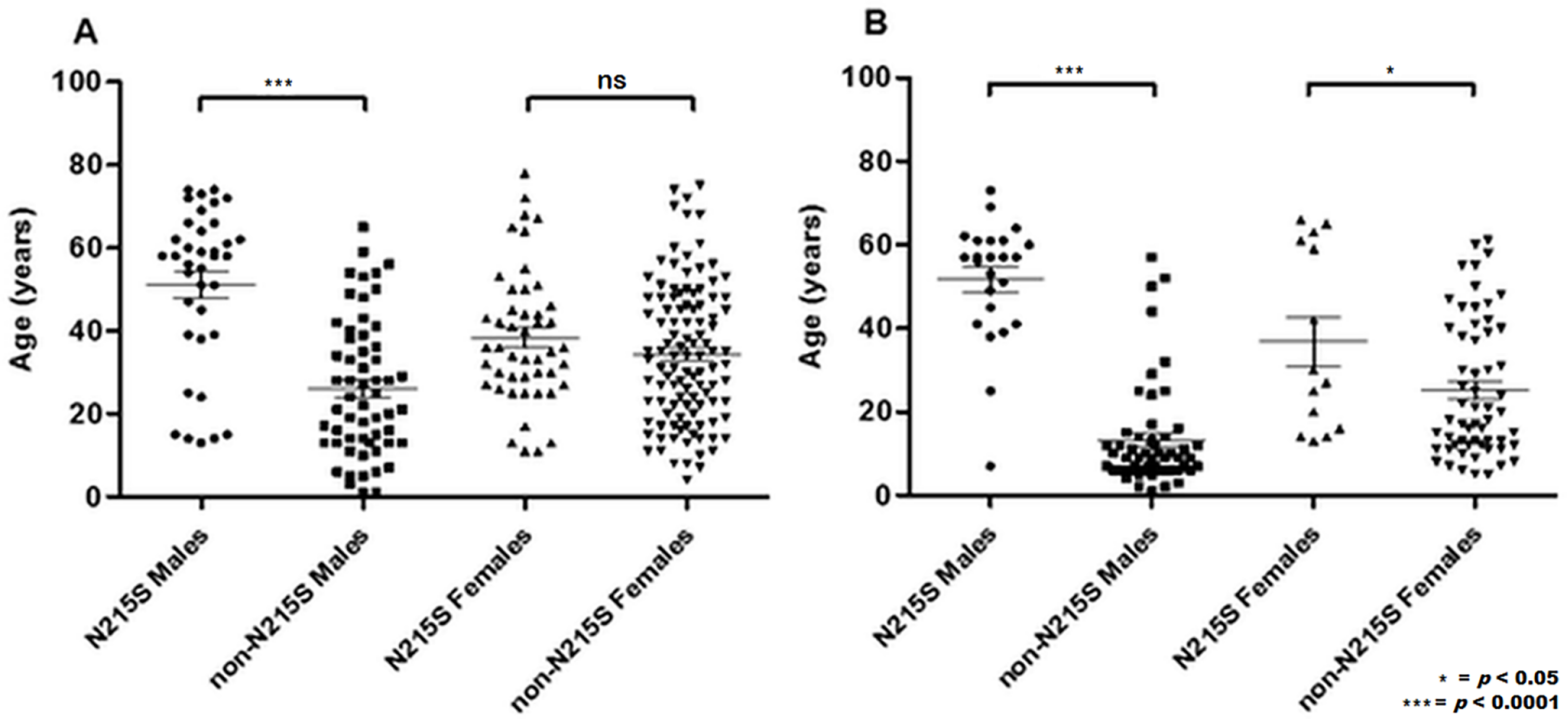
(A) Age at diagnosis for males *N215S* (n = 37), males *non-N215S* (n = 58) and females *N215S* (n = 47), females *non-N215S* (n = 109). (B) Age at symptom onset for males *N215S* (n = 24), males *non-N215S* (n = 53) and females *N215S* (n = 14), females *non-N215S* (n = 59); ns = not significant.

*N215S* index males had a significantly older age of symptom onset compared to those with *non-N215S* mutations (median age 57 years vs. 9 years, p<0.0001, Fig 1B), as well as age of diagnosis (median age 58 years vs. 23 years, p<0.0001; Fig 1A).

First signs and symptoms among index cases included: (1) Cardiac: shortness of breath, palpitations, syncope, abnormal ECG, chest pain, fatigue, murmur, cardiomegaly found on screening, and cardiac arrest. (2) Renal: proteinuric CKD, haematuria, hypertension, renal failure, proteinuria during pregnancy, and persistent proteinuria post pregnancy. (3) Dermatological: rash and angiokeratomas. (4) Neurological: pain, migraine, and TIA. (5) Strokes: single episodes and recurrent strokes. (6) Ophthalmological: sore eye, cornea verticillata, and eye deposits. (7) Gastrointestinal: irritable bowel syndrome (IBS) (Table 1).

The *N215S* mutation presented predominately with later onset cardiac manifestations in males. In 18 out of 22 index *N215S* patients the initial disease manifestations were cardiac, either symptomatic (syncopal episodes, palpitations or chest pain) or the incidental finding of LVH during pre-operative assessments or insurance medicals (7 patients) (Table 1). In two *N215S* index males the initial symptoms were of IBS, with the diagnosis being made following subsequent development of cardiac manifestations. Two *N215S* male index cases presented at a significantly younger age (25 and 38 years) with ESRF. In contrast, acroparesthesia and/or angiokeratoma were the commonest initial symptoms in *non-N215S* index males, occurring in 17 out of 21 patients (81%, p<0.0001). In three the initial symptoms were cardiac (onset aged 32, 44, and 50 years) and one proteinuria (initially noted age 29).

Both groups attained similar overall severity scores, but at different ages. Mild *N215S* patients (MSSI < 20) were significantly older compared to *non-N215S* (mean age: 36.6, n = 19 vs. 29.5 yeas, n = 6, p = 0.005). Moderate (MSSI = 20–40) and severe (MSSI≥ 40) patients were also older in the *N215S* group (63.7 years (n = 17) vs. 35.6 years (n = 45) p< 0.0001 and 71 (n = 1) vs. 29.7 years (n = 2), p = ns, respectively).

Females accounted for over 50% of index cases in the *non-N215S* population compared with only two (8.3%) in *N215S* patients (p = 0.0004), one presenting with proteinuria in pregnancy aged 25 years and the other with cardiac hypertrophy aged 68. Whilst *non-N215S* women were diagnosed, on average, 8.4 years after males (means: 34.5 vs. 26.1 years, p = 0.002), *N215S* women were diagnosed 12.8 years before males with the same mutation (means: 38.3 vs. 51.1 years, p< 0.002) due to family screening of younger generations. Only *non-N215S* females reached severe overall MSSI severity scores (MSSI ≥ 40), both at baseline and during follow-up (Table 1).

Whilst similar proportions of male patients were on ERT (*N215S*: 33/37, 89.2% vs. *non-N215S*: 56/58, 96.6%), only 11/47, 23.4% of *N215S* females were on treatment compared to 62/109, 56.9% in the *non-N215S* group (p = 0.0001). Indeed, *N215S* females received the least proportion of any concomitant medication when compared to the rest of the cohort (Table 2).

**Table 2:** 

Parameters	N215S		non-N215S	
Overall	N = 84		N = 167	
	Males	Females	Males	Females
N (%)	37 (44.1)	47 (55.9)	58 (34.7)	109 (65.3)
**Risk factors for cardiovascular disease**				
Smoking history, n (%)	15 (40.5)	8 (17)	7 (12.1)	19 (17.4)
Obesity (≥35 km/m2), n (%)	3 (8.1)	1 (2.1)	-	5 (4.6)
Diabetes mellitus, n (%)	5 (13.5)	3 (6.4)	1 (1.7)	3 (2.8)
**Fabry related symptoms**				
**Genera**l				
Hypertension, n (%)	13 (35.1)	7 (14.9)	8 (13.8)	19 (17.4)
Tortuous vessels, n (%)	3 (8.1)	2 (4.3)	14 (24.1)	15 (13.8)
Acroparestesia, n (%)	8 (21.6)	7 (14.9)	47 (81)	51 (46.8)
Angiokeratoma, n (%)	9 (24.3)	5 (10.6)	44 (75.9)	33 (30.3)
**CNS**				
Brain imaging changes (MRI / CT), n (%)	19 (51.4)	13 (27.7)	29 (50)	40 (36.7)
Cerebrovascular event, n (%)	3 (8.1)	1 (2.1)	11 (19)	16 (14.7)
**Cardiac**				
LVH, n (%)	17 (46)	4 (8.5)	22 (37.9)	24 (22)
Arrhythmia, n (%)	11 (29.7)	0 (0)	3 (5.2)	13 (11.9)
Conduction abnormalities, n (%)	11 (29.7)	8 (17)	10 (17.2)	17 (15.6)
LVMI, n (missing)	31 (6)	40 (7)	49 (9)	82 (27)
Mean (SD)	58.2 (22.7)	33 (16.3)	53.1 (20.2)	42.7 (15.9)
Median (range)	50.6 (27.6–105.3)	29.3 (9.8–94.4)	48.6 (23.2–129)	38 (18.2–94.6)
Remodelling, n (missing)	25 (12)	31 (16)	35 (26)	68 (41)
Normal1, n (%)	7 (28)	24 (77.4)	14 (40)	43 (63.2)
Concentric2, n (%)	5 (20)	4 (12.9)	9 (25.7)	7 (10.3)
Concentric hypertrophy3, n (%)	11 (44)	3 (9.7)	10 (28.6)	16 (23.5)
Eccentric hypertrophy4, n (%)	2 (8)	-	2 (5.7)	2 (2.9)
Permanent pacemaker, n (%)	7 (18.9)	1 (2.1)	2 (3.5)	1 (0.9)
Implantable cardioverter-defibrillator, n (%)	6 (16.2)	0 (0)	3 (5.2)	2 (1.8)
**Renal**				
Proteinuria, n (%)	12 (32.4)	3 (6.4)	23 (39.7)	21 (19.3)
CKD stages, n (missing)	37 (0)	41 (6)	49 (9)	99 (10)
1(> 90 mL/min/1.73m2)	16	27	25	57
2(60–89 mL/min/1.73m2)	13	13	16	32
3A(45–59 mL/min/1.73m2)	4	0	4	5
3B(30–44 mL/min/1.73m2)	1	1	3	4
4 (>15–29 mL^/^min/1.73m2)	1	-	1	1
5(<15 mL/min/1.73m2 or on dialysis)	2	-	-	-
**Concomitant medication**				
*ARB*/ACE therapy any time, n (%)	17 (46)	6 (12.8)	27 (46.6)	33 (30.3)
ß blocker therapy any time, n (%)	12 (32.4)	3 (6.4)	6 (10.4)	15 (13.8)
Antiplatelet therapy any time, n (%)	19 (51.4)	9 (19.2)	22 (37.9)	32 (29.4)
Anticoagulant therapy any time, n (%)	4 (10.8)	0 (0)	3 (5.2)	8 (7.3)
Statins therapy any time, n (%)	20 (54.1)	8 (17)	15 (25.9)	25 (22.9)

Anticoagulant therapy includes warfarin; antiplatelet therapy includes aspirin, dipyridamole, and clopidogrel; Brain imaging changes were assessed through magnetic resonance imagining (MRI) and computed tomography (CT), they include white matter lesions (WML), infarcts, vessels dissection and tortuosity. CNS = central nervous system; LVH = left ventricular hypertrophy, assessed through echocardiogram (>48 g/m^2.7^ in females and >50 g/m^2.7^ in males); CKD = chronic kidney disease; LVMI = left ventricular mass indexed to height. 1. Normal LVMI + RWT ≤ 42%. 2. Normal LVMI + RWT ≥ 42%. 3. Increased LVMI + RWT ≥ 42%. 4. Increased LVMI + RWT ≤42%. [36]. Proteinuria: >150 g/24hs. Arrhythmia included Wolf-Parkinson-White syndrome, supraventricular tachycardia, atrial fibrillation, ventricular fibrillation, non-sustained ventricular tachycardia, ventricular tachycardia and paroxysmal atrial fibrillation. Conduction abnormalities included long QT, short PR and conduction blocks.

### Follow up and survival

**Fig 2:**
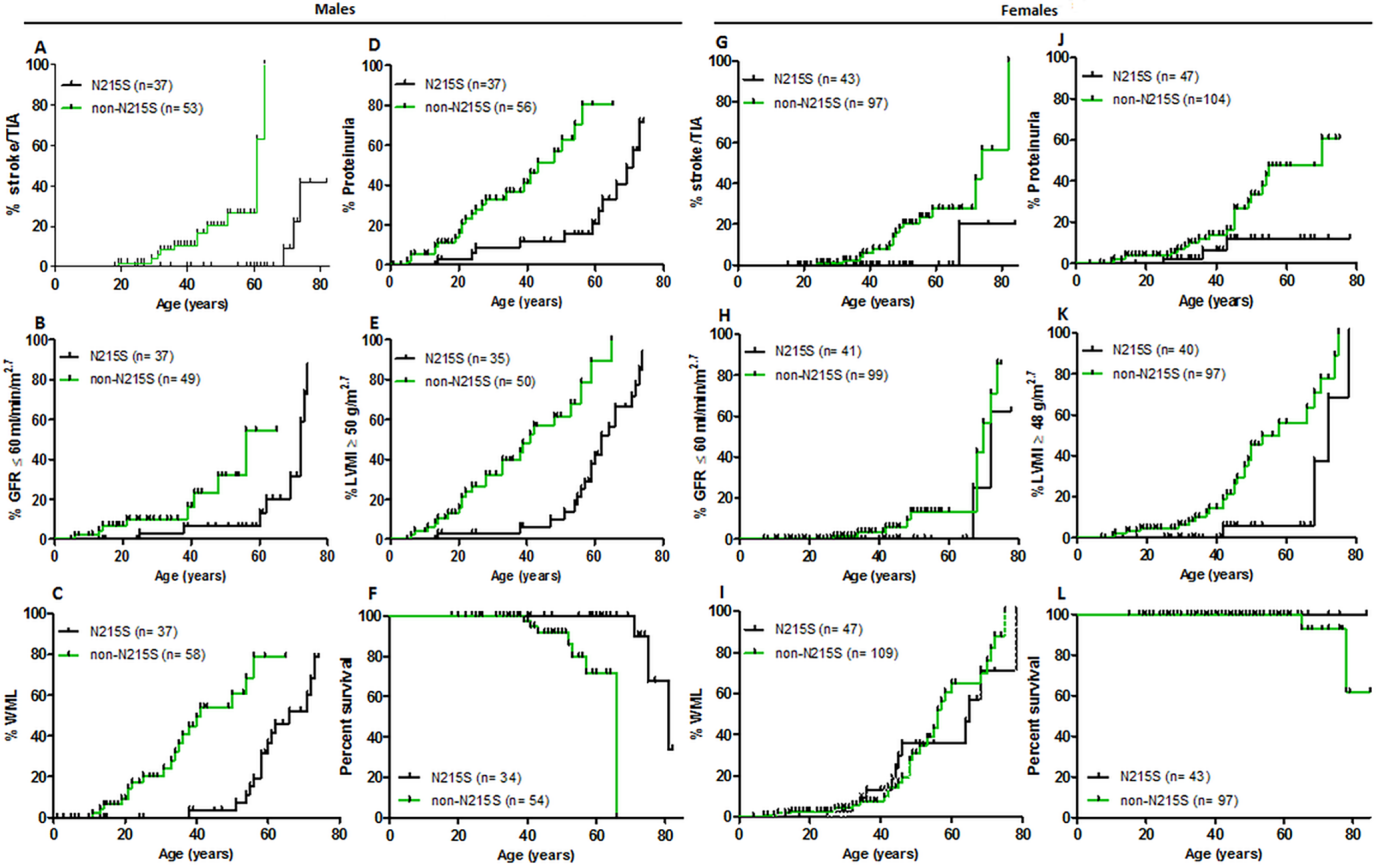
Kaplan-Meier survival analyses. A-F Males; G-L Females. (A, G) Stroke or Transient ischaemic attack (TIA), (B, H) stage III chronic kidney disease (GFR <60ml/min/1.73m2), (C, I) White matter lesions (WML), (D, J) Proteinuria (defined as a protein excretion of over 150g/24hs), (E, K) Left ventricular mass by echocardiogram indexed to height (LVMI) ≥ 50g/m2.7 in males and ≥ 48g/ m2.7 in females, and (F, L) Overall survival, during follow-up, by gender and mutation status. The small vertical lines represent censored data (follow-up until the vertical line without development of an event).

Baseline assessments were performed on a total of 95 males (index and screening) at a mean age of 26.1 years in *non-N215S* males (n = 58) and 51.1 years in *N215S* (n = 37), p <0.0001. The mean age at latest follow up for *non-N215S* males was 44.1 years (range 20–65, n = 51) and 56.3 years for *N215S* males (range 18–82, n = 35). There were 10 deaths amongst male FD patients, 7 in *non-N215S* males (3 cardiac related, 3 stroke and 1 malignancy; mean age 48.3 years) and 3 *N215S* males (all cardiac; mean age 75.7 years). Only 2 females passed away during the follow up, one carrying the *G361R* mutation (cardiac death, aged 77) and the other harboured a *H46Y* mutation (she had a stroke at 65 years old). Overall, survival was significantly greater in *N215S* males than *non-N215S* males (Fig 2F, median survival 81 vs. 66 years, p< 0.0006). However, we did not find significant differences in overall survival amongst females (Fig 2L).

### Cardiac manifestations (Table 2)

Cardiac evaluation at baseline included ECG and echocardiogram (LVH defined as an LVMI > 48 g/m^2.7^ in females and > 50 g/m^2.7^ in males). 29 patients were missing echocardiogram at baseline (9 males, 20 females).

24 (28.6%) *N215S* patients (20 males, 4 females) and 49 (29.3%) *non-N215S* (23 males, 26 females) had LVH on echocardiogram. 1 male *non-N215S* had LVH by voltage criteria (LVMI data missing). Furthermore, 2 male *N215S* patients and 3 *non-N215S* (1 male, 2 females) were found to have LVH at baseline but their LVMI data was not available.

Of the 6 patients one presented with ESRF at age 25, two teenagers had conduction abnormalities on ECG and showed no symptoms, 1 required an ICD after developing a Non-Sustained Ventricular Tachycardia (NSVT) with pre-syncope, and 1 had acroparesthesia, gastrointestinal symptoms and a few WML on brain MRI. Only 4 *N215S* females had developed LVH at baseline and they were aged 68, 72, 78 and 42 years old. Cardiac hypertrophy was present in all except for 8 male *non-N215S* patients (*R301Q* (aged 14), *A143T* (aged 1), *P205T* (aged 3), *exon 1 deletion* (aged 13), *c*.*700_702 del GAT deletion* (aged 27), *G361R* (aged 11), *L372P* (aged 19), and *R49L* (aged 28)). The patients with *c*.*700_702 del GAT deletion*, *L372P*, and *R49L* have subsequently developed conduction abnormalities. However, only 30/109 of *non-N215S* female patients exhibited cardiac hypertrophy, at a mean age of 43.3 (range 10–75) years old.

**Fig 3:**
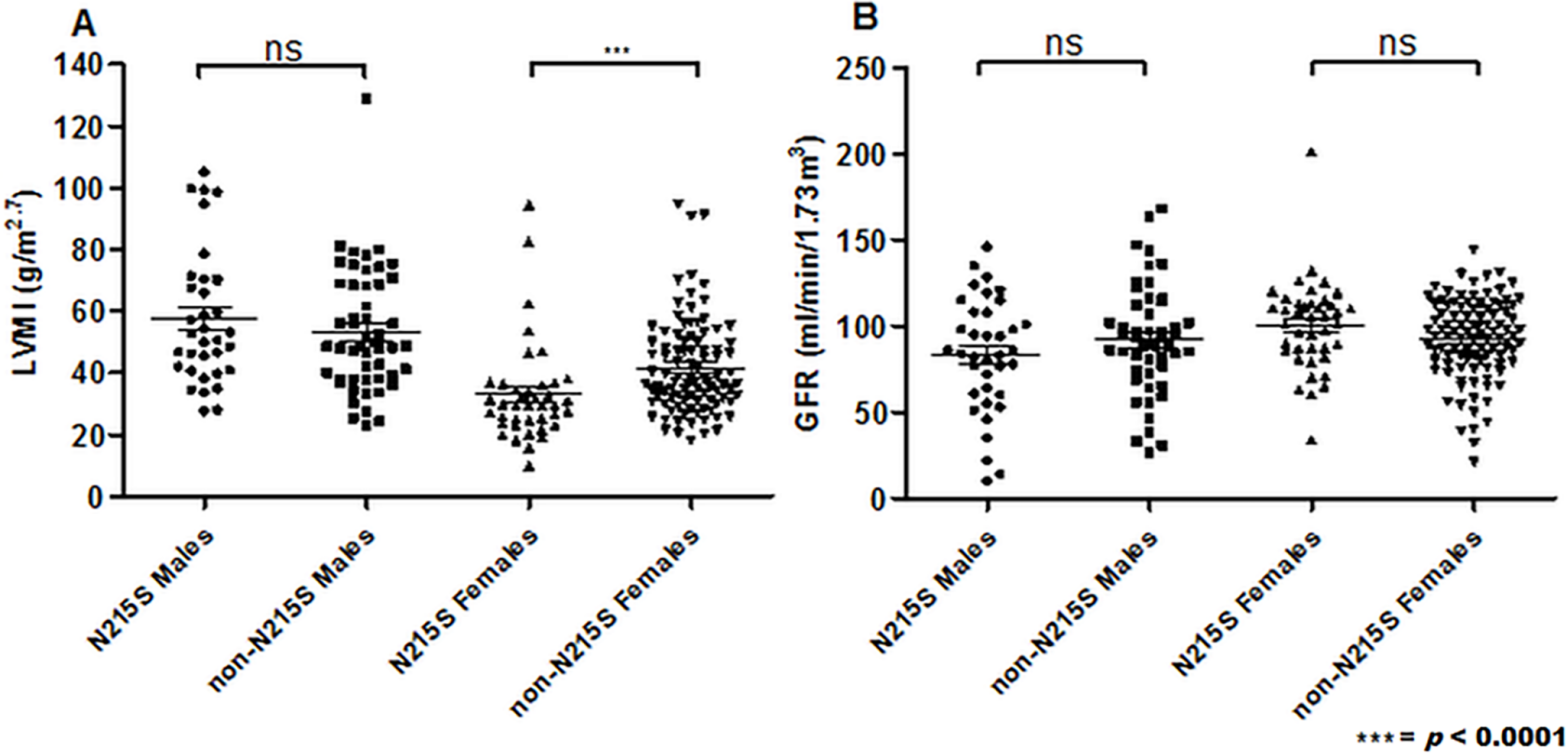
Quantification of: (A) Left ventricular mass indexed to height (LVMI) for males *N215S* (n = 35), males *non-N215S* (n = 51) and females *N215S* (n = 40), females *non-N215S* (n = 96). (B) Glomerular filtration rate (GFR) for males *N215S* (n = 37), males *non-N215S* (n = 50) and females *N215S* (n = 41), females *non-N215S* (n = 99).

Most males exhibited concentric hypertrophy with no difference between mutation groups (Table 2). On echocardiogram, there were no differences in LVMI magnitude between mutation groups (Fig 3A). However, Kaplan-Meier survival analysis demonstrated that the development of a LVMI over 50g/m^2.7^ in males occurred at a younger age for the *non-N215S* population (median survival: 41 vs. 64 years, p< 0.0001, Fig 2E). Females described a similar disposition (50 vs. 72 years, p = 0.003) (Fig 2K). 13 (29.7%) *N215S* men have had ICD or permanent pacemakers (PPM) inserted for arrhythmias at a median age of 69 years (range 51–79), whereas only 5 (8.8%) *non-N215S* men required one at a median age of 56 years (range 47–59, Table 2). Only 4 women required an ICD or PPM inserted, 1 *N215S* aged 63 years old and 3 *non-N215S* (aged 60, 64, and 72 years old, Table 2).

As potential contributory factors, 15 (40.5%) *N215S* men had smoking history compared to only 7 (12.1%) classic men. Furthermore, 5 (13.5%) *N215S* men had type 2 diabetes compared to only 1 (1.8%) *non-N215S* man (Table 2). In the case of females, similar proportions had smoking history, 6.4% of *N215S* and 2.8% of *non-N215S* are diabetic and, of note, 5 *non-N215S* patients had a BMI over 35 kg/m^2^ compared to only 1 *N215S* woman (Table 2). Hypertension was more common in *N215S* males (35.1% vs. 13.8%, p<0.03 Table 2), however, in females it predominated in the *non-N215S* group (17.4% vs. 14.9%, p = not significant, Table 2).

Beta blockers and statins therapies were more prevalent amongst *N215S* males, whereas angiotensin converting enzyme inhibitors (ACEI) / angiotensin receptor blockers (ARB) were in *non-N215S* males. However, for females, all of these four concomitant medications were more common in the *non-N215S* population (Table 2).

### Renal manifestations

Similar proportions of *non-N215S* (23/58, 39.7%) and *N215S* (12/37, 32.4%) males had proteinuria at baseline (Table 2), but proteinuria developed at an earlier age in *non-N215S* males (median survival without development of proteinuria: 43 vs 71 years, p< 0.0001, Fig 2D). Two *N215S* males with proteinuria had diabetes as a potential contributory factor compared to only 1 *non-N215S* man (Table 2). There were no significant differences in GFR at baseline (Fig 3B), yet, survival curve analysis revealed that stage II CKD (GFR <60ml/min/1.73m2) is developed at a significantly younger age in non-*N215S* males (median survival: 56 vs. 72 years, p< 0.01, Fig 2B). Progression to stage 5 CKD (GFR <15ml/min/1.73m2) occurred in 2 *N215S*, all of whom had marked persistent proteinuria.

The three index males whose initial disease manifestations were renal (2 *N215S*, 1 *non-N215S*) have all had or are awaiting renal transplants. One further *non-N215S* male patient was diagnosed on family screening having been symptomatic with acroparesthesia since age 7, had a GFR of 33 at diagnosis aged 34 and has progressed to end stage renal disease (ESRD). The other *N215S* patient was diagnosed on family screening aged 73, but had a history of proteinuria of undefined aetiology first detected at age 56 and a partial nephrectomy many years previous for tuberculosis.

Amongst females, proteinuria was more common in the *non-N215S* group (19.3% vs. 6.4%, p = not significant, Table 2) and Kaplan-Meier analysis demonstrated that it also develops earlier in this group (median survival: 70 years vs. not reached -*N215S*-, p = 0.03, Fig 2J). Furthermore, there were no differences on GFR at baseline between mutation groups (Fig 3B). *Non-N215S* females also developed stage II CKD (GFR <60ml/min/1.73m2) at an earlier age but this age difference was not significant (70 vs. 72 years old, Fig 2H).

### Cerebrovascular manifestations

Cerebrovascular events (stroke/TIA) were more common in *non-N215S* males (11/58, 19%) than *N215S* males (3/37, 8.1%, p = not significant, Table 2) and occurred at a younger age (median survival: 61 years vs. not reached -*N215S*-, p< 0.0001, Fig 2A). In the case of females, *non-N215S* had more cerebrovascular events (stroke/TIA) compared to *N215S* (14.7% vs. 2.1%, p< 0.03, Table 2) and these occurred earlier in life (median survival: 74 years vs. not reached -*N215S*-, p< 0.02, Fig 2G). Cerebral WML were found in similar proportions of *non-N215S* (29/58, 50%) and *N215S* (19/37, 51.4%) males but were found at a significantly younger age in *non-N215S* (median age 44 vs. 66 years, p<0.0001, Fig 2C). Females described a comparable distribution to males, but with no significant differences amongst mutation groups (Table 2, Fig 2I).

### Relationship of disease manifestations to enzyme activity

Males with the *N215S* mutation had a significantly lower baseline age-adjusted FOS-MSSI severity score (mean score -5 vs. +7.6, p<0.0001) and higher leukocyte α-Gal A activity (7.0 vs 2.32 nmol/mg protein/hr, p< 0.0001). They also showed a trend towards higher plasma enzyme activity compared to *non-N215S* (0.25 vs. 0.17 nmol/ml/hr, p = 0.07).

**Fig 4:**
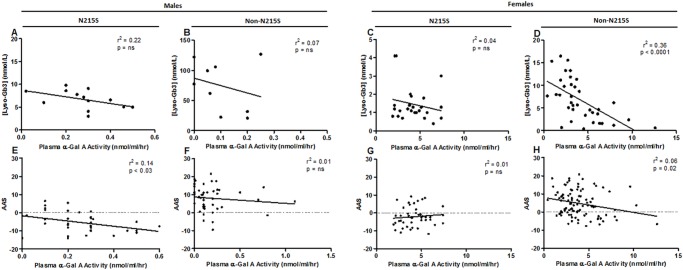
Correlation analyses: (1) Plasma globotriaosylsphingosine (Lyso-Gb3) and plasma α-Gal A activity for males *N215S* (A; n = 13), males *non-N215S* (B; n = 9), females *N215S* (C; n = 25), and females *non-N215S* (D; n = 36). (2) Age adjusted severity score (AAS) and plasma α-Gal A activity for males *N215S* (E; n = 36), males *non-N215S* (F; n = 48), females *N215S* (G; n = 45), and females *non-N215S* (H; n = 98); ns = not significant.

**Fig 5:**
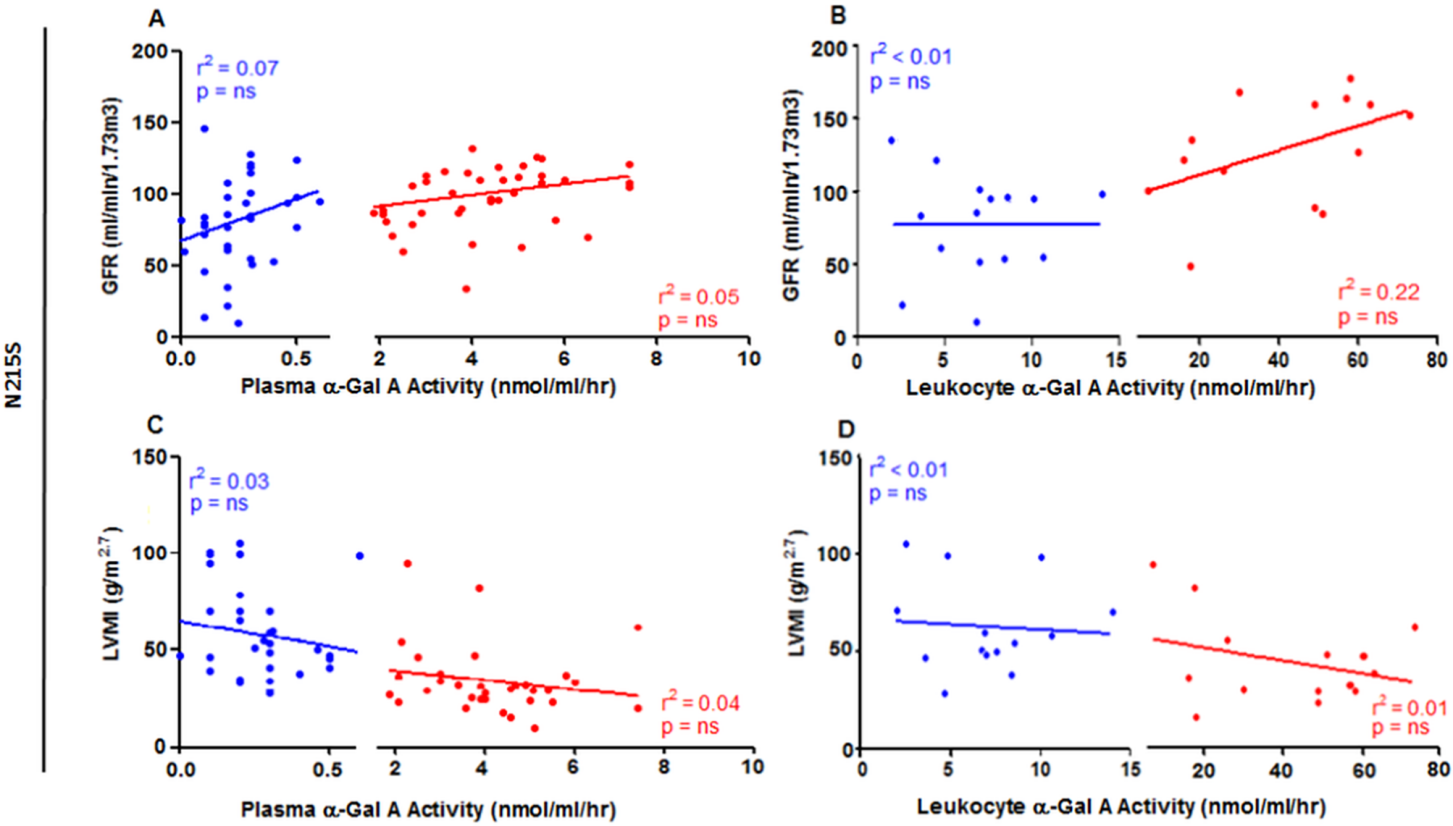
Correlation analyses: red = females; blue = males: (A) Glomerular filtration rate (GFR) and plasma α-Gal A activity for males (n = 36) and females (n = 40). (B) GFR and leukocyte α-Gal A activity for males (n = 15) and females (n = 14). (C) Left ventricular mass indexed to height (LVMI) and plasma α-Gal A activity for males (n = 30) and females (n = 32). (D) LVMI and leukocyte α-Gal A activity for males (n = 14) and females (n = 14); Data shown for *N215S* group (non-*N215S*: S1 Fig); ns = not significant.

Despite the same *GLA* mutation, there was marked variation in enzyme activity amongst *N215S* patients, with enzyme activity correlating weakly with overall disease severity (r^2^ = 0.136, p<0.03, n = 36, see Fig 4E), but not with LVMI or GFR (Fig 5 and S1 Fig).

α-Gal A activity was higher in females regardless of *GLA* variant (means: 4.04 vs 0.21 nmol/mg protein/hr, p< 0.0001) and demonstrated weak correlation with severity in the *non-N215S* females only (data not shown).

### Ageing

There were no significant correlations for neither gender, between either leukocyte or plasma α-Gal A activity and age (S2 Fig). In univariate regression analyses, we found that MSSI significantly correlated with age, LVMI and GFR for the *N215S* patients but not the *non-N215S* patients (Table 3).

**Table 3:** Univariate linear regression analyses.

		N215S		non-N215S	
		Univariate analysis		Univariate analysis	
	Lyso-Gb3]	r^2^ = 0.61	p< 0.003	r^2^ = 0.15	p = ns
**MSSI**	Age	r^2^ = 0.73	p = 0.0002	r^2^ = 0.13	p = ns
	[Lyso-Gb3]*age	r^2^ = 0.88	p< 0.0001	r^2^ = 0. 35	p = ns
	[Lyso-Gb3]	r^2^ = 0.52	p< 0.01	r^2^ = 0.05	p = ns
**LVMI**	Age	r^2^ = 0.40	p< 0.03	r^2^ = 0.25	p = ns
	[Lyso-Gb3]*age	r^2^ = 0.59	p< 0.005	r^2^ = 0.02	p = ns
	[Lyso-Gb3]	r^2^ = 0. 33	p< 0.05	r^2^ < 0.01	p = ns
**GFR**	Age	r^2^ = 0.74	p = 0.0002	r^2^ = 0.25	p = ns
	[Lyso-Gb3]*age	r^2^ = 0.75	p = 0.0001	r^2^ = 0.26	p = ns

MSSI = Mainz Severity Score Index; GFR = Glomerular filtration rate; LVMI = left ventricular mass indexed to height; Lyso-Gb3 = Globotriaosylsphingosine (nmol/L); Age is expressed in years; [LysoGb3]*age = Life exposure to Lyso-Gb3 (nmolyr/L).

### Plasma Globotriaosylsphingosine (lyso-Gb3)

Lyso-Gb3 was found to be elevated in both *N215S* and *non-N215S* compared with historical controls for both males and females (Table 4). At baseline, in untreated patients, lyso-Gb3 was higher in the *non-N215S* males and females than *N215S* males and females, respectively. Female *non-N215S* lyso-Gb3 was also higher than male or female *N215S*. Significant heterogeneity was noted in lyso-Gb3 levels in all groups (Table 4). On average, lyso-Gb3 was lower in the ERT- treated group for both *N215S* and others compared to their untreated counterparts (Table 4). Lyso-Gb3 levels were significantly lower in both, treated (5.8 vs 28.9 nmol/L, p< 0.0001) and untreated (4.6 vs. 34.4 nmol/L, p< 0.004) women, regardless of *GLA* variant (data not shown).

**Table 4:** Plasma Lyso-Gb3 quantification.

	N215S		non-N215S	
Overall	N = 84		N = 167	
	Males	Females	Males	Females
N (%)	25 (43.1)	33 (56.9)	39 (23.4)	72 (43.1)
**Lyso-Gb3 (nmol/L)**				
*ERT naïve*, n (%)	13 (52)	26 (78.8)	9 (23.1)	36 (50)
Mean (SD)	6.7 (2.1)	1.5 (0.9)	74.3 (42.2)	6.8 (4.6)
Median (range)	6.6 (3–9.8)	1.2 (0.4–4.1)	77.4 (20.9–126.9)	6 (0.4–16.5)
*During ERT*, n (%)	12 (48)	7 (21.2)	30 (76.9)	36 (50)
Mean (SD)	6.3 (3.3)	1.7 (1.5)	37.9 (21.3)	6.7 (3.4)
Median (range)	4.9 (2.6–13.6)	1.1 (0.4–4.9)	33.8 (1.4–113.2)	5.9 (0.4–16.7)

Lyso-Gb3 = Globotriaosylsphingosine. Upper reference limit Lyso-Gb3 = 0.6.

**Fig 6:**
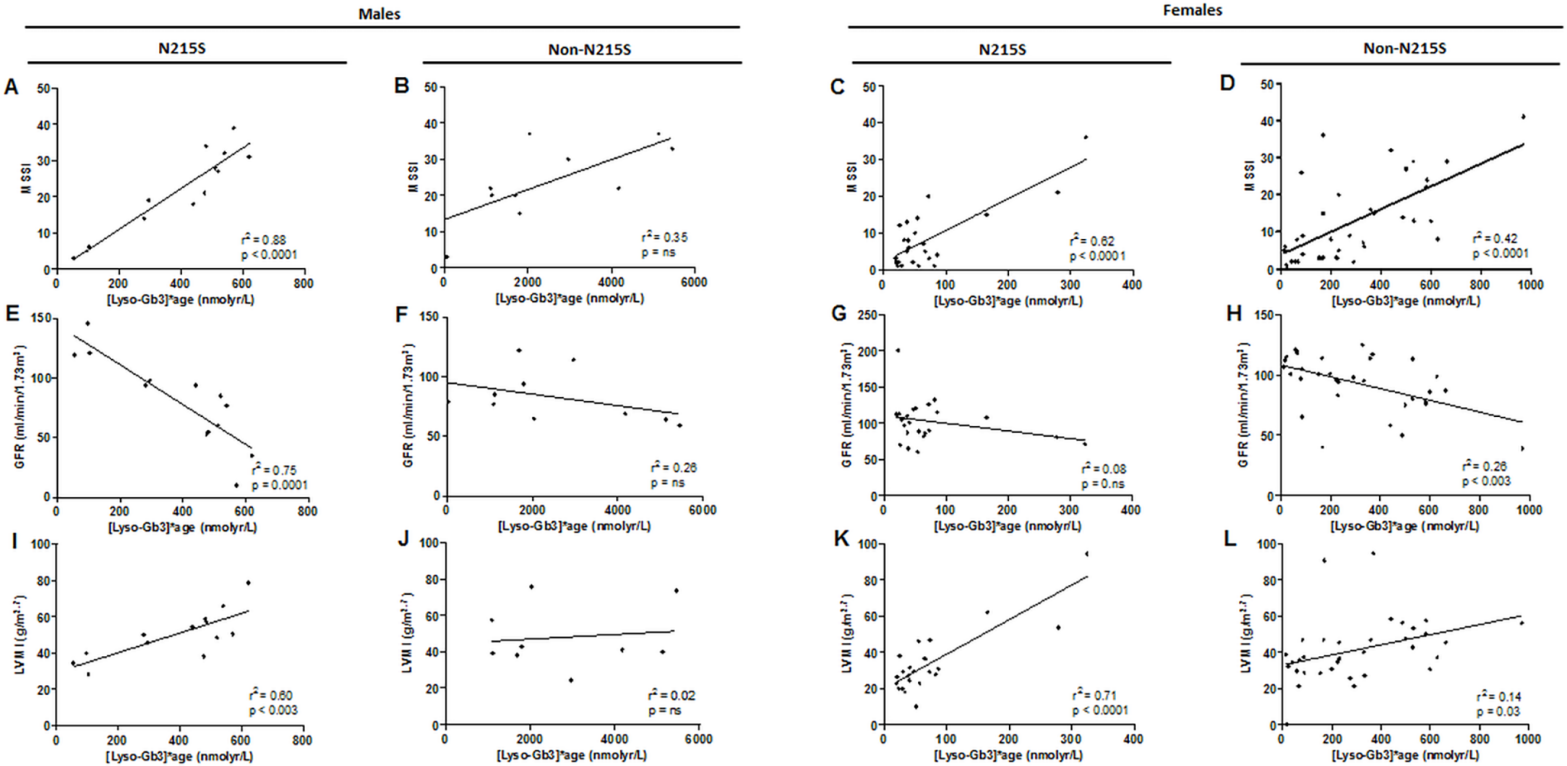
Correlation analyses: Life time exposure to Lyso-Gb3 ([Lyso-Gb3]*age) and: clinical severity, (MSSI) for: (A) *N215S* males (n = 13), (B) *non-N215S* males (n = 9), (C) *N215S* females (n = 26), and (D) *non-N215S* females (n = 36); baseline GFR for: (E) *N215S* males (n = 13), (F) *non-N215S* males (n = 9), (G) *N215S* females (n = 26), and (H) *non-N215S* females (n = 33); LVMI for: (I) *N215S* males (n = 12), (J) *non-N215S* males (n = 9), (K) *N215S* females (n = 23), and (L) *non-N215S* females (n = 34); ns = not significant.

The *N215S* population reached an overall severity measured by MSSI comparable to the *non-N215S* without equivalent elevation of lyso- Gb3 (Fig 6A–6D).

**Fig 7:**
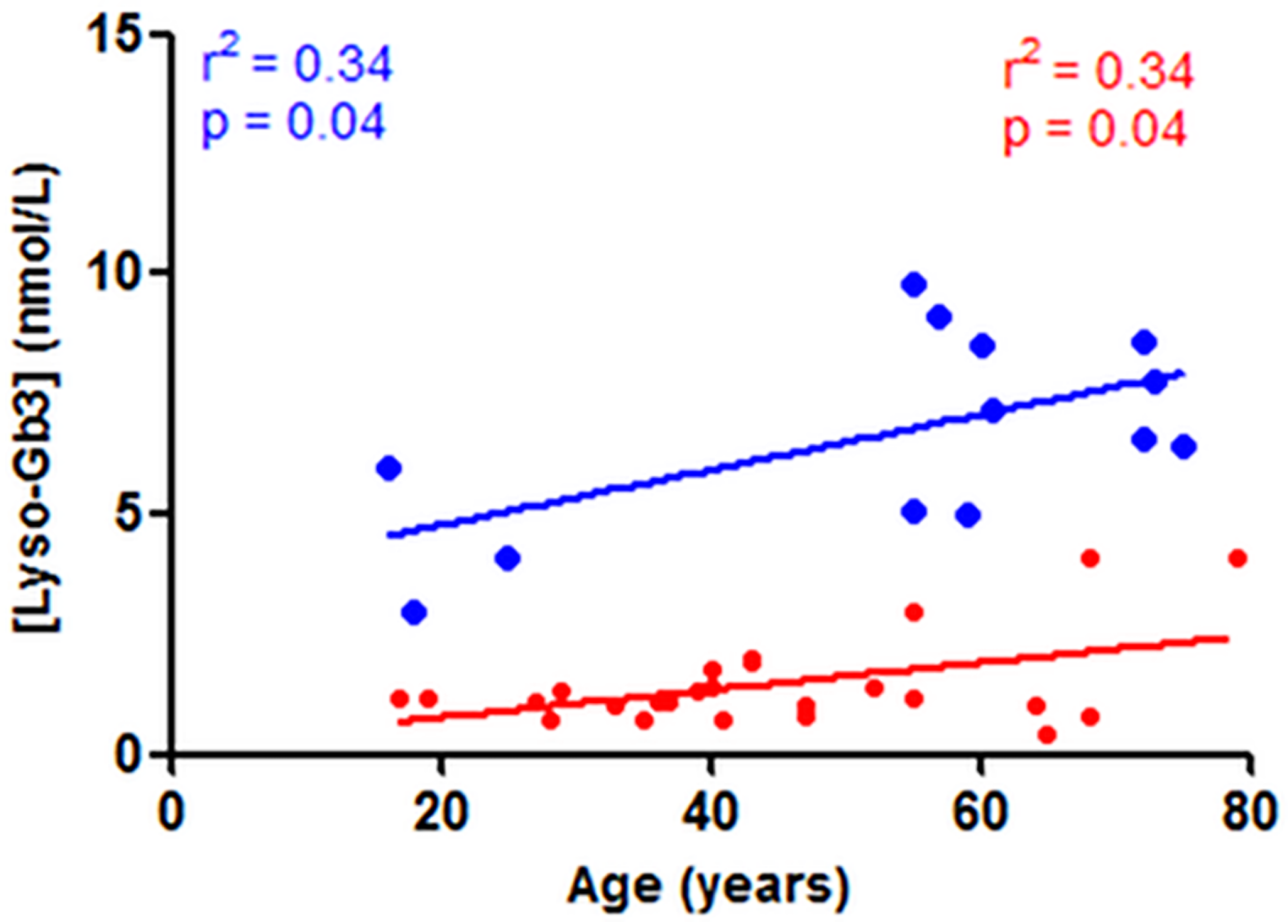
*N215S* correlation analyses: Plasma globotriaosylsphingosine (Lyso-Gb3) and age for males (blue; n = 13) and females (red; n = 26).

Older male *N215S* patients showed higher plasma lyso-Gb3 levels, and exhibited a significant association between plasma lyso-Gb3 levels and age (r^2^ = 0.34, p = 0.04, Fig 7), not seen for the non-*N215S* group (S3 Fig). Conversely, lower levels of plasma lyso-Gb3 were found in the non-*N215S* patients in the same age group (Fig 7 and S3 Fig) likely due to a survivor effect or milder forms. Both age and lyso-Gb3 demonstrated a significant correlation with MSSI, LVMI and GFR (26, 27) (Table 3). However, in univariate linear regression analyses, life time exposure to lyso-Gb3 levels (calculated as [lyso- Gb3]*age) showed a stronger significant positive correlations with MSSI (r2 = 0.88, p< 0.0001, Fig 6A) and LVMI (r2 = 0.59, p< 0.005, Fig 6I), and a negative one with GFR (r2 = 0.75, p = 0.0001, Fig 6E). No similar correlations were seen for the *non-N215S* group (Fig 6B, 6F and 6J).

In the case of females, we found strong correlations in the *N215S* group between life time exposure to lyso-Gb3 and MSSI (r^2^ = 0.62, p< 0.0001, Fig 6C) and LVMI (r^2^ = 0.71, p< 0.0001, Fig 6K). However, the *non-N215S* group showed significant but weak correlations between life time exposure to lyso-Gb3 and the clinical parameters (MSSI: r^2^ = 0.42, p< 0.0001, Fig 6D; GRF: r^2^ = 0.26, p< 0.003, Fig 6H; LVMI: r^2^ = 0.14, p = 0.03, Fig 6L). No significant correlation was noted between plasma lyso-Gb3 and α-Gal A activity, plasma (Fig 4) or leukocyte (data not shown).

**Fig 8:**
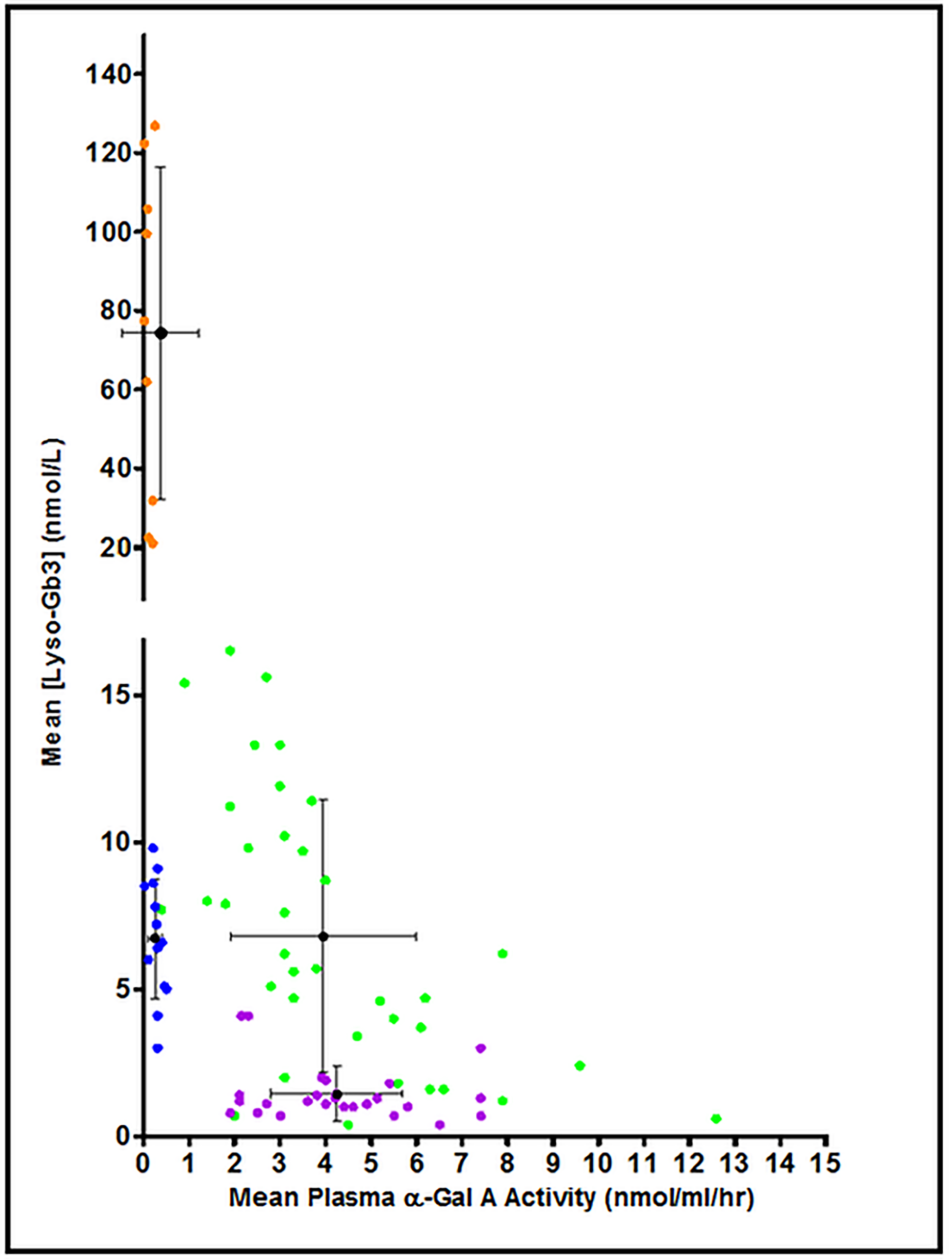
Correlation analyses: Plasma globotriaosylsphingosine (Lyso-Gb3) and plasma α-Gal A activity for males *N215S* (blue; n = 13), males *non-N215S* (orange; n = 9) and females *N215S* (purple; n = 26), females *non-N215S* (green; n = 36).

Through this analysis four groups could be distinguished (Fig 8) with distinct relationships between plasma lyso-Gb3 and plasma enzyme activity, for example males who despite not showing a significant difference in enzyme activities had different plasma lyso-Gb3 concentrations (means: 6.7 vs. 74.3 nmol/L, p < 0.001, Table 4).

## Discussion

The diagnosed prevalence of Fabry disease in the UK has increased substantially over the last 17 years since enzyme replacement therapy became available. This is in part due to raised awareness and screening of high risk populations, including those found to have unexplained left ventricular hypertrophy. In this cohort of 251 Fabry patients, 33.5% (84) were found to have the *N215S* mutation, which has been described in association with a later onset cardiac phenotype. Many of the pertinent index cases were referred from cardiology clinics with subsequent diagnosis of family members due to cascade screening, however, some patients presented in ESRF. At present it is unclear if these pedigrees are distinct or in fact one family as a result of a founder effect in the UK. Further haplotype analysis is required to confirm this.

We have compared the clinical and biochemical phenotype of this group of patient to the rest of our cohort who harbour *non-N215S* mutations in the *GLA* gene. 59 different mutations comprised the latter mutation group including deletions, nonsense, and missense mutations (see S1 Table). *N215S* patients exhibited a distinct phenotype with significantly later symptom onset than patients with other mutations. Primary presenting symptoms were cardiac disease but in some cases renal failure. Males and females displayed higher levels of leukocyte enzyme activity and lower levels of lyso-Gb3 than *non-N215S* counterparts. Kaplan-Meyer analysis revealed that *N215S* patients develop cardiac hypertrophy and proteinuria at an older age, and have a greater overall survival. Indeed, *N215S* men exceeded the general population life expectancy for over 1.9 years, whereas *non-N215S* patients was reduced compared to the general population by 13.1 years [37]. This fact might explain some of the severity found in the *N215S* population, who might acquire more disease burden as a result of ageing.

Patients who harboured *non-N215S* mutations, and in particular those associated with the classical phenotype, mainly presented with acroparesthesias and angiokeratomas and rarely cardiac or renal involvement. They also showed an earlier decline in renal function to GFR <60 ml/min/1.73m^2^ and an earlier occurrence of cerebrovascular events (stroke/TIA).

There were no differences in LVMI between mutation groups at baseline, however, at the time of the first assessment the mean age was 25 years older in the *N215S* males. *N215S* males reached similar total MSSI scores to that of *non-N215S* males but, on average, 25.5 years later. Males with *N215S* attained similar overall MSSI scores and degree of LVMI to *non-N215S* males despite higher residual enzyme activity and lower plasma lyso-Gb3 levels. Liao et al. reported similar differences in lyso-Gb3 levels between classical and late onset patients, and similar associations between lyso-Gb3, age and LVMI in their Taiwanese patients carrying the late onset mutation IVS4 + 919G>A [38]. This may suggest that the heart is more sensitive than other organs to the effects of lyso-Gb3. With manifestations occurring at lower levels of lyso-Gb3 in patients with relatively higher leucocyte activities of α-Gal A, lyso-Gb3 may play a direct role in FD pathogenesis. However, storage alone cannot explain the myocardial remodelling found on these patients [39]. Other studies have suggested a pro-proliferative effect of lyso-Gb3 on smooth muscle cells [3] and a cumulative effect on human podocytes [40], inducing the expression and production of extracellular matrix proteins via TGF-β1 [41] and Notch1 pathway [42].

Plasma lyso-Gb3 levels appeared to be useful to differentiate between phenotypes in males, however *N215S* males and classical females had similar lyso-Gb3 levels [21, 43]. Despite having on average only 9% plasma lyso-Gb3 of the *non-N215S* patients, the plasma lyso-Gb3 levels of *N215S* males showed strong correlations with LVMI, GFR and MSSI. Indeed, these associations became even stronger when we investigated the individual’s life time exposure to lyso-Gb3, considering the impact of age.

Our results demonstrated that both age and lyso-Gb3 are major factors influencing *N215S* patients’ clinical outcomes and overall disease severity (measured by MSSI), LVMI and GFR. Furthermore, for *N215S* patients, strong significant correlations of clinical manifestations with life time exposure to lyso-Gb3 suggests that the cumulative exposure is crucial for the disease progression. Contrary to previous suggestions [23, 44] this finding supports the utility of lyso-Gb3 and its age related function in prediction of disease severity in newly diagnosed late onset patients.

None of the correlations between plasma lyso-Gb3 and clinical manifestations found in *N215S* patients were evident for *non-N215S* male patients, consistent with the results of Rombach et al. who investigated these associations in 37 classic male Fabry patients [43]. The absence of these associations might indicate that above certain levels of lyso-Gb3 in plasma, higher concentrations are not predictive of the degree of disease severity.

Lower levels of plasma lyso-Gb3 were found in treated patients, implying an effect of ERT; however, this is a cross-sectional analysis and could reflect a treatment bias therefore further longitudinal studies are required.

*N215S* index cases were mostly males, and women were generally diagnosed through cascade screening. Regardless of the mutation, female patients showed higher enzyme activity, lower lyso-Gb3 levels, and received, proportionally, less treatment (either ERT or concomitant medications). The ratio of females to males in our *N215S* cohort is only 1.3 to 1 which is lower than the expected 2 to 1 for an X-linked disease, and may suggest that *N215S* female patients are underdiagnosed due to their mild phenotype. Even in classic men clinical diagnosis of FD can be difficult due to frequent misinterpretation of early symptoms, and often patients present with a long consultation history. Hsu et al. reported that cardiac disease can progress silently, without showing significant clinical symptoms [45]. Family screening is therefore key to identifying asymptomatic individuals in order to provide appropriate genetic counselling prognostication and monitoring.

## Strengths and limitations

This is a cross sectional baseline study of a large number of patients with a single mutations compared to other patients without that mutation in our cohort all investigated and managed according to a common protocol. We identify a number of strengths including the standardised follow up and completeness of the genotyping and clinical data. A further strength is that we have chosen to compare the *N215S* cohort of patients to our total cohort of *non-N215S* patients including the full range of genotypes some of which are also late onset thereby avoiding the bias of selecting only mutations previously described to have classic phenotype. We report data prior to the initiation of therapy for clinical parameters in all patients however a limitation is that lyso-Gb3 at baseline was only available for a subset in which the analysis is performed. Similarly, whilst genotyping was available in all patients a full profile of enzyme activities was not available for every patient. For the small number of patients diagnosed prior to 1999 there is a lapse in time between date of diagnosis and baseline assessment as patient were not immediately seen in the specialist centre.

We present event and survival follow up for the genotype-defined cohorts as a whole and have not further analysed by exposure to enzyme replacement therapy which was outside of the scope of this study. After baseline assessment similar proportions of male *N215S* patients v *non-N215S* patients were in receipt of ERT although a smaller proportion of *N215S* females were on treatment reflecting their overall milder phenotype which would have been a confounder in any analysis of ERT response. Similarly, the sample size lacked the necessary power to detect statistically significant differences in ERT effect by genotype.

According to our local protocols, during the majority of the follow up period echocardiography with its potential limitations of variability rather than MRI was used to measure LVMI.

## Conclusion

Patients with the *N215S* mutation exhibit a distinct phenotype with later onset but not exclusively cardiac manifestations, with some patients presenting with renal disease. Contrary to previous reports, lyso-Gb3 is an important parameter in the assessment of patients with the *N215S* mutation. In particular severity and clinical manifestations correlated with life time exposure to plasma lyso-Gb3 in the *N215S* group and may prove useful in prognostication and therapeutic decision making.

## Supporting information

S1 Fig Correlation analyses. (A-D) GFR and: (a) plasma α-Gal A activity for males *non-N215S* (A; n = 42) and females *non-N215S* (B; n = 94), (b) leukocyte α-Gal A activity for *non-N215S* males (C; n = 28) and *non-N215S* females (D; n = 44). (E-H) LVMI and: (a) plasma α-Gal A activity for males (E; n = 44) and *non-N215S* females (F; n = 76), (b) leukocyte α-Gal A activity for *non-N215S* males (G; n = 29) and *non-N215S* females (H; n = 42); ns = not significant.
(TIF)

S2 Fig Correlation analyses. plasma α-Gal A activity and age for males: (A) *N215S* (n = 36), (B) *non-N215S* (n = 49), and females: (C) *N215S* (n = 46), *non-N215S* (n = 102); ns = not significant.
(TIF)

S3 Fig Correlation analyses. Plasma globotriaosylsphingosine (Lyso-Gb3) and age for Non-*N215S* males (blue; n = 9) and *non-N215S* females (red; n = 36); ns = not significant.
(TIF)

S1 Table Mutations included in the non-N215S group. (PDF)
