# Supplementary figures and images for: Phenotype and biochemical heterogeneity in late onset Fabry disease defined by N215S mutation
Source: PLoS One. 2018 Apr 5;13(4):e0193550. doi: 10.1371/journal.pone.0193550 (PMC5886405; doi:10.1371/journal.pone.0193550)

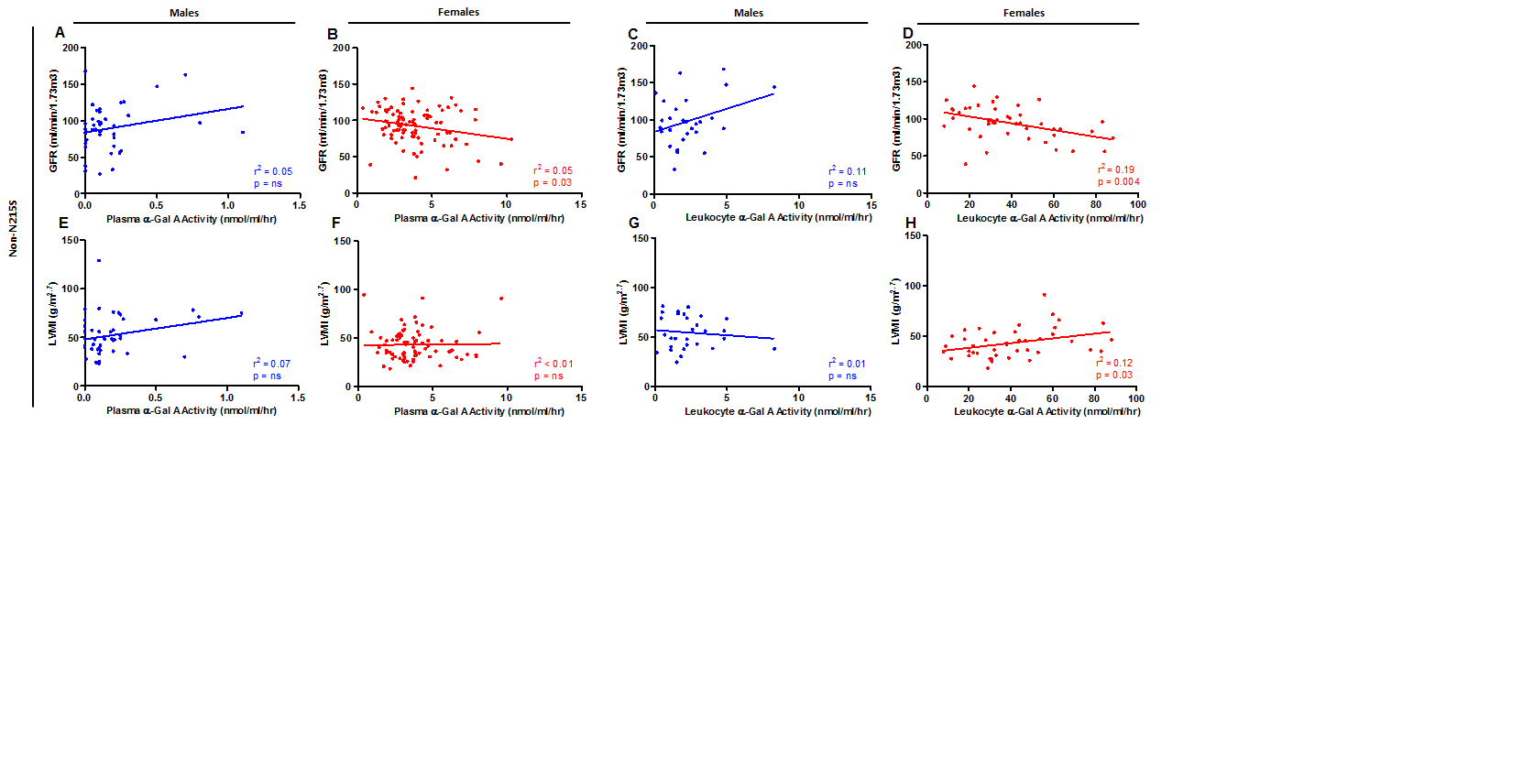

Supplement: S1 Fig — (A-D) GFR and: (a) plasma α-Gal A activity for males non-N215S (A; n = 42) and females non-N215S (B; n = 94), (b) leukocyte α-Gal A activity for non-N215S males (C; n = 28) and non-N215S females (D; n = 44). (E-H) LVMI and: (a) plasma α-Gal A activity for males (E; n = 44) and non-N215S females (F; n = 76), (b) leukocyte α-Gal A activity for non-N215S males (G; n = 29) and non-N215S females (H; n = 42); ns = not significant. (TIF) [file pone.0193550.s001.tif]

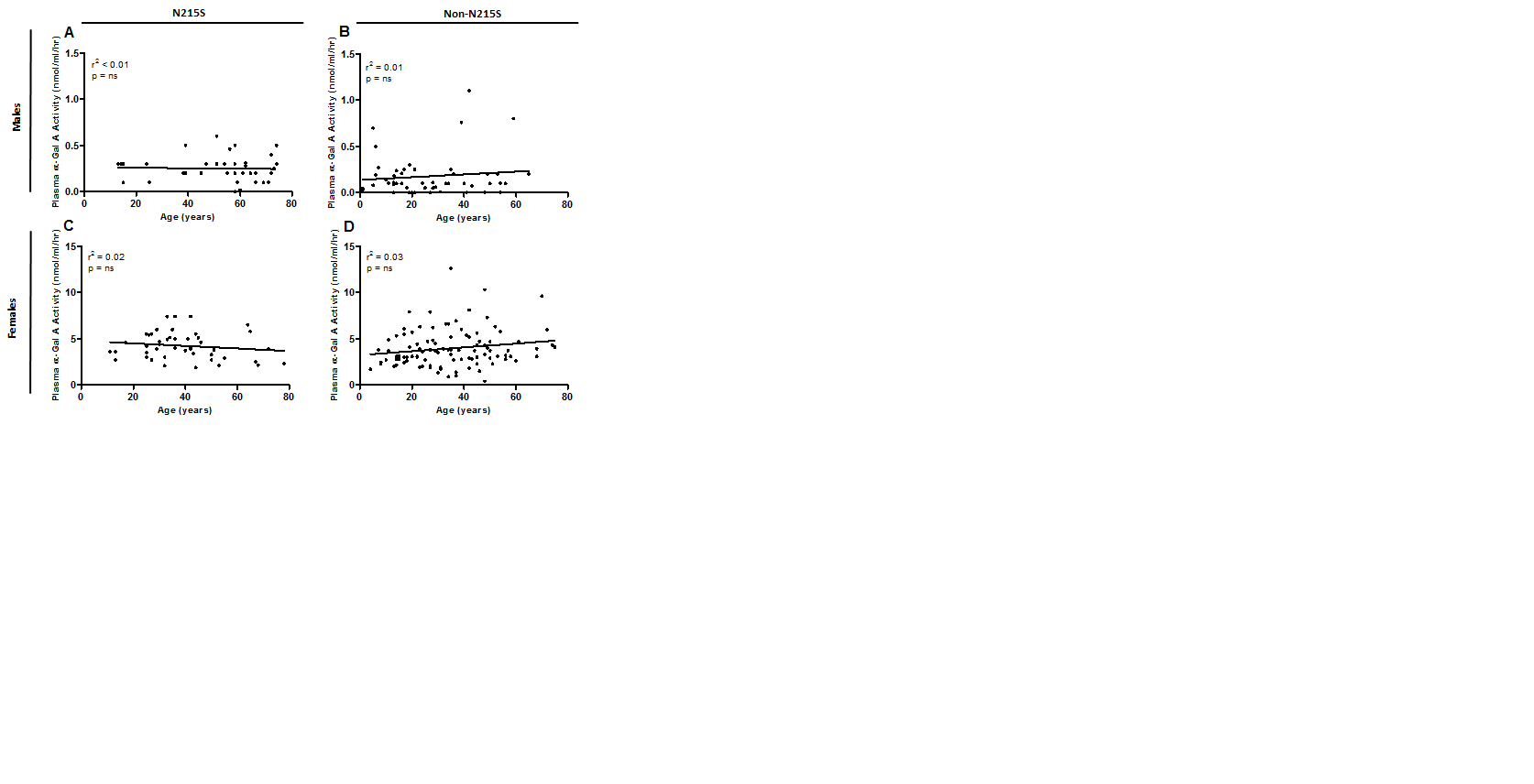

Supplement: S2 Fig — plasma α-Gal A activity and age for males: (A) N215S (n = 36), (B) non-N215S (n = 49), and females: (C) N215S (n = 46), non-N215S (n = 102); ns = not significant. (TIF) [file pone.0193550.s002.tif]

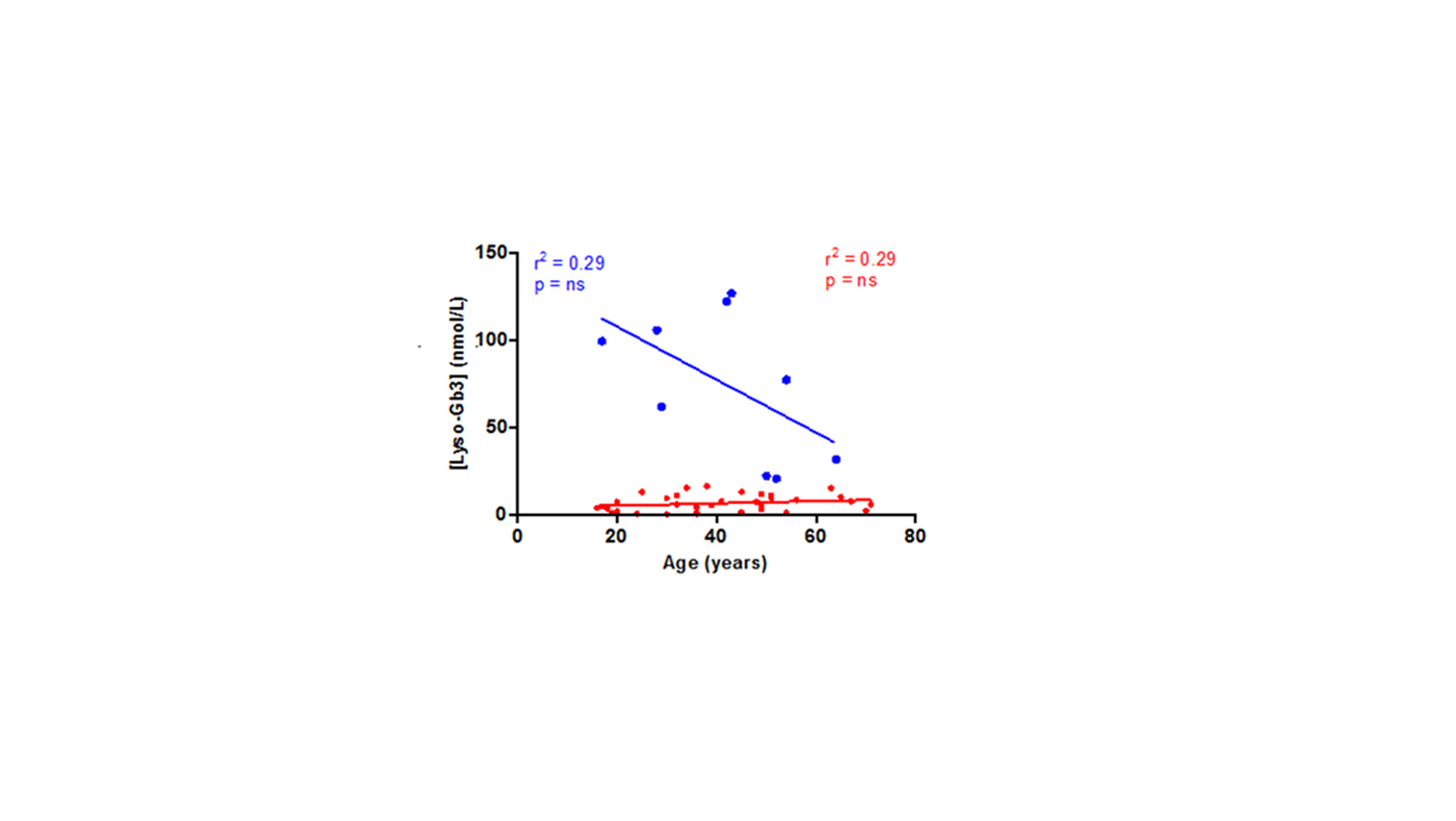

Supplement: S3 Fig — Plasma globotriaosylsphingosine (Lyso-Gb3) and age for Non-N215S males (blue; n = 9) and non-N215S females (red; n = 36); ns = not significant. (TIF) [file pone.0193550.s003.tif]
