# Supplementary material for: Phenotype and biochemical heterogeneity in late onset Fabry disease defined by N215S mutation
Source: PLoS One. 2018 Apr 5;13(4):e0193550. doi: 10.1371/journal.pone.0193550 (PMC5886405; doi:10.1371/journal.pone.0193550)
Supplement: S1 Table — (PDF) [file pone.0193550.s004.pdf]

Supplemental table 1
Mutations included in the non-N215S group.

Mutations	Number of patients
Missense	
A13P	2
A143T	9
A257P	1
A309P	2
A31V	3
C52G	2
D93Y	1
E338K	5
G261D	1
G261V	1
G361A	3
G361R	3
H46Y	1
I303N	1
I317T	9
I91T	1
L166P	5
L372P	2
M42V	2
N33D	3
P205T	13
P293H	2
P409T	3
Q221P	1
Q279H	1
Q280H	2
R112C	1
R112H	2
R301P	1
R301Q	9
R342Q	3
R49L	2
S126G	1
T410I	2
T410L	1
V269A	2
V316E	2

Mutations	Number of patients
Nonesense	
Q107X	2
R220X	1
R227X	20
R301X	2
S78X	1
W209X	1
W227X	1
W277X	2
Y184X	2
Small deletions and other rearrangements	
c.1223del6	1
c.359_364 del TAGCTAdel	2
c.402 del T	1
c.466delG	2
c.520delT	8
c.700_702 del GAT del	3
c.717-718 het del AA	1
c.717del2	1
c.748_801+8del62	2
c.802-3_802-2 delCA	3
exon 1 deletion	3
exon 7 deletion	1
c.589het-dupA	1
